# Utilization of SiC and Cu Particles to Enhance Thermal and Mechanical Properties of Al Matrix Composites

**DOI:** 10.3390/ma12172770

**Published:** 2019-08-28

**Authors:** Dongxu Wu, Congliang Huang, Yukai Wang, Yi An, Chuwen Guo

**Affiliations:** School of Electrical and Power Engineering, China University of Mining and Technology, Xuzhou 221116, China

**Keywords:** composites, Al powder, SiC nanoparticle, thermal conductivity, compressibility

## Abstract

In this work, SiC and Cu particles were utilized to enhance the thermal and mechanical properties of Al matrix composites. The ball-milling and cold-compact methods were applied to prepare Al matrix composites, and the uniform distribution of SiC and Cu particles in the composite confirms the validity of our preparation method. After characterizing the thermal conductivity and the compressibility of the prepared composites, results show that small particles have a higher potential to improve compressibility than large particles, which is attributed to the size effect of elastic modulus. The addition of SiC to the Al matrix will improve the compressibility behavior of Al matrix composites, and the compressibility can be enhanced by 100% when SiC content is increased from 0 to 30%. However, the addition of SiC particles has a negative effect on thermal conductivity because of the low thermal conductivity of SiC particles. The addition of Cu particles to Al-SiC MMCs could further slightly improve the compressibility behavior of Al-SiC/Cu MMCs, while the thermal conductivity could be enhanced by about 100% when the Cu content was increased from 0 to 30%. To meet the need for low density and high thermal conductivity in applications, it is more desirable to enhance the specific thermal conductivity by enlarging the preparation pressure and/or sintering temperature. This work is expected to supply some information for preparing Al matrix composites with low density but high thermal conductivity and high compressibility.

## 1. Introduction

Compared with conventional materials, metal matrix composites (MMCs) have higher specific strength, specific modulus, and light weight, among other advantages. In the preparation of MMCs, aluminum (Al) is an ideal matrix candidate because of its low melting point, high specific strength, and high thermal conductivity [1,2,3,4,5]. Al-based MMCs have been widely used in the aircraft, military, aerospace, and automotive industries [6,7]. Owing to the low density, low melting point, high specific strength and thermal conductivity of Al, a wide variety of reinforcement particles, including ceramic particles (SiC, Al_2_O_3_), metal particles (Cu, Mg) and graphite, have been used to reinforce it in order to further improve its mechanical and physical properties [8,9,10,11,12,13]. Kollo et al. [14] reported that the ultimate tensile strength of the Al-based MMCs could increase from 205 MPa to 420 MPa with an increase in the volume fraction of SiC nanoparticles from 1 to 10%. Similar findings were also reported in Refs [15,16]. Choi et al. [17] found that the addition of Cu could also greatly enhance the hot-tearing resistance of Al-based MMCs. Zhao et al. [18] studied the effect of graphene on the mechanical properties of Al/graphene MMCs, which were able to achieve a Vickers microhardness of 120 HV. Hamedan et al. [19] prepared 1.0 wt.% nano-sized SiC/Al MMCs and reported that the ultimate tensile strength of the MMC increased from 140 MPa to 173 MPa, but that the ductility decreased slightly from 6.1% to 5.38%.

Although a lot of work has been focused on the mechanical properties of Al-based MMCs [19,20,21,22], the understanding of the effects of reinforcements on thermal conductivity of Al-based MMCs is still lacking, and the thermal conductivity is also an important consideration in applications [23,24,25,26], such as in the heat dissipation of electric devices. A low or an inhomogeneous thermal conductivity in MMCs may cause catastrophic failures as a result of overheating or deformation [27,28,29,30]. The thermal conductivity of the Al-based MMC can be greatly influenced by volume fractions, distributions and thermal conductivities of reinforcements. In addition to thermal conductivity, the compressibility of MMCs is also an important property when they are used in the aircraft, military, aerospace and automotive industries [31], yet there is still a scarcity of works focusing on the influence of reinforcements on the compressibility of MMCs [1,32].

In this work, the effect of the addition of nanometers and micrometers of SiC and Cu on the thermal conductivity and compressibility of Al MMCs was studied. High-energy ball milling was used to prepare the Al MMC powder, and then the Al MMC powder was pressed into MMCs by cold compaction, which was able to produce the widest range of powder metallurgy engineering materials for studying the compressibility of the Al MMCs. The hot-wire method was used to measure the thermal conductivity of the Al MMCs in order to study the effect of SiC and Cu additions on thermal conductivity. Finally, methods to increase the specific thermal conductivity of Al MMCs are proposed.

## 2. Experiment

**Figure 1:**
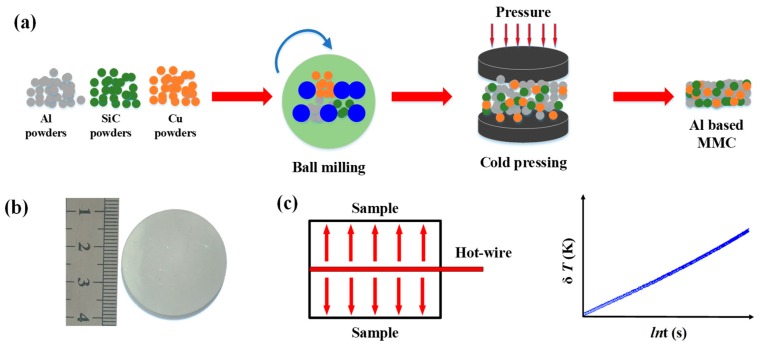
MMC preparation and thermal conductivity measurements: (**a**) schematic diagram of Al-based MMC preparation; (**b**) macro view of Al-based MMCs; (**c**) hot-wire method for thermal conductivity measurement.

Two different diameters (500 nm and 1 μm) of Al, β-SiC (SiC) and Cu particle powders commercially obtained from Beijing DK Nano Technology Co., Ltd were used as raw materials for MMC preparation. The sizes of the particles were estimated from the SEM image using ‘Image J’ software, and the estimated sizes follow a Gaussian distribution, as shown in Appendix A. Based on the figures, the average size can be easily calculated, and this also matches well with the size supplied by the particle provider. Given that high-energy ball milling is a common technique for attaining a homogeneous distribution of reinforcements in MMCs [33,34,35,36], this method was also applied in this work to prepared Al MMCs. In the preparation process, as illustrated in Figure 1a, raw powders with the same diameter were firstly milled for 6 hours to form Al-SiC composites with different SiC contents (0, 10, 20 and 30 wt%) and Al-30%SiC/Cu composite with designed Cu content (0, 20 and 30 wt%) in a vertical planetary ball mill with a speed of 200 rpm using 5-mm-diameter corundum balls. The ball-to-powder weight ratio was set as 10:3 in the milling process. Then, the milled powders were consolidated by cold uniaxial pressing to form 3-cm-diameter cylindrical Al MMCs. Different pressures were applied to obtain different porosities of Al MMCs, such as 10, 20, 30, 40 and 50 MPa. For each pressure, three samples were prepared and the average values of relative densities and thermal conductivities were obtained. The prepared Al-based MMCs are shown in Figure 1b.

The microstructures of the Al MMCs were observed by field-emission scanning electron microscopy (SEM) on a Quanta SU-8820 instrument (Hitachi co., Tokyo, Japan) with an acceleration of 10 KV. The thermal conductivities of Al MMCs were measured using a commercial device (Model TC3000, Xian XIATECH Technology, Xi’an, China), and were established based on the hot-wire method. The working principle of the hot-wire method is schematically shown in Figure 1c. In this measurement, the hot wire is sandwiched between two pieces of the same sample, and then the temperature is obtained by measuring the electrical resistance of the hot-wire. After obtaining the temperature–time curve, as shown in Figure 1c, the thermal conductivities of samples can easily be calculated by [37,38]

(1) $$k=\frac{q}{4\pi }\frac{lnt_{2}-lnt_{1}}{T_{2}-T_{1}}$$

where *q* is the electric heating power per unit length, and $T_{1}$ and $T_{2}$ are the temperatures at times $t_{1}$ and $t_{2}$, respectively. The instrumental error was less than 3%, and more details about this method can be found in our previous works [39,40,41].

The relative density of Al MMCs can be obtained by the mass-volume method. The mass was measured by an electronic balance with a measurement accuracy of 0.0001 g, and the volume was calculated by the cross-sectional area multiplied by the thickness, where the cross-sectional area is equal to that of the mold, and the thickness was measured using a spiral micrometer with an accuracy of 0.001 mm. For each sample, 10 different measurements were taken to calculate an average thickness. The relative density of MMCs can be calculated by dividing the bed density by the density of the raw particles, where the density of Al was taken to be 2.7 × 10^3^ kg/m^3^, SiC was taken to be 3.2 × 10^3^ kg/m^3^, and Cu was taken to be 8.9 × 10^3^ kg/m^3^. The porosity uncertainty was less than 0.001 in this work.

## 3. Results and Discussions

Given the important influence of particle distribution in MMC structures on compressibility and thermal conductivity, the particle distributions in the MMCs are firstly characterized in Section 3.1. After confirming the uniform distribution of particles in the MMCs, the effects of the reinforcement content and the compacting pressures on compressibility and thermal conductivity are further discussed.

### 3.1. Structural Characterization

**Figure 2:**
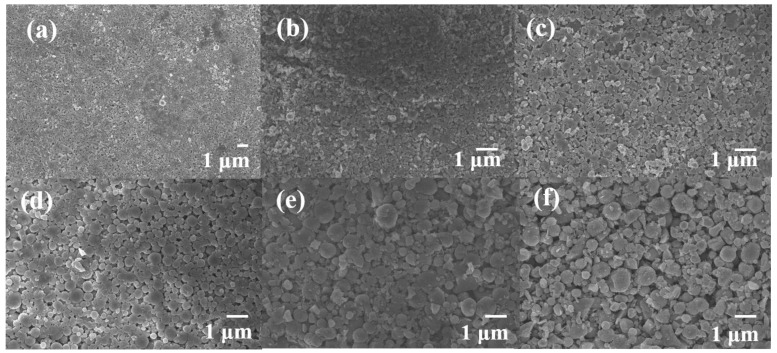
Microstructures of Al MMCs prepared with 10 MPa pressure: (**a**) 500-nm Al MMCs; (**b**) 500-nm Al-30%SiC MMCs; (**c**) 500-nm Al-30%SiC/30%Cu MMCs; (**d**) 1-μm Al MMCs; (**e**) 1-μm Al-30%SiC MMCs; (**f**) 1-μm Al-30%SiC/30%Cu MMCs.

**Figure 3:**
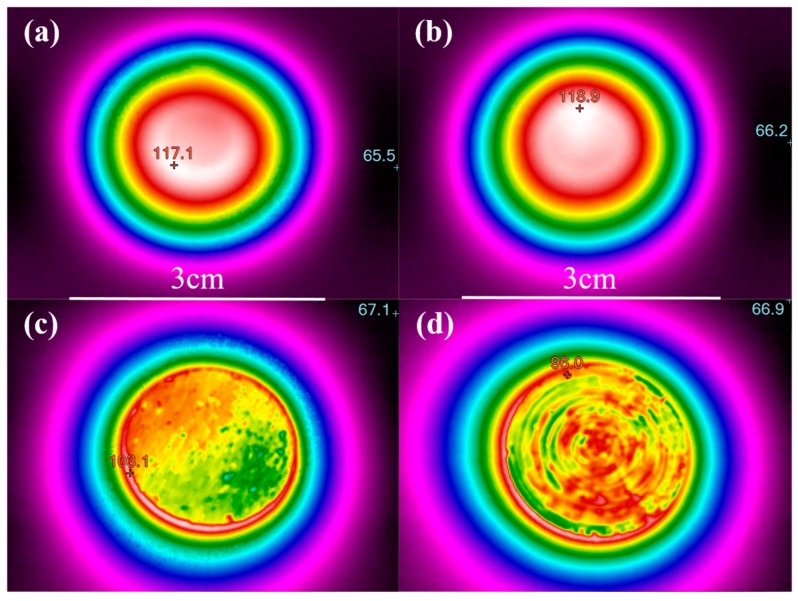
Temperature distributions in Al MMCs: (**a**) 500-nm Al-30%SiC MMC prepared with our method; (**b**) 500-nm Al-30%SiC/30%Cu MMCs prepared with our method; (**c**) 500-nm Al-30% SiC MMCs with short-time milling for comparison; (**d**) 500-nm Al-30%SiC/30% Cu MMCs with short-time milling for comparison.

The diameters and the contents of the raw materials used in the preparation of the Al MMCs are respectively shown in Table 1 and Table 2, respectively. The same sizes of Al, SiC and Cu particles were used in the preparation of Al MMCs in this work. Microscopic views of Al MMCs after ball-milling are shown in Figure 2, and microscopic views of the raw particles are shown in Appendix B. It can be seen that SiC nanoparticles are able to adhere to Al particles with a relatively uniform distribution. In our ball milling process, the shapes of the Al and Cu particles were not changed and remained near-spheroidal, while the SiC particles were fractured and became finer due to this material’s high hardness and deformation resistance [42]. The distributions of SiC and Cu in the Al MMCs were further characterized with IR images. Different samples were firstly heated to a temperature of 150 °C, and then placed on a piece of wood to cool naturally. After several seconds of cooling, IR images of different samples were taken, as shown in Figure 3. Figure 3a,b shows the temperature distributions of Al-based MMCs prepared using our previously introduced method, while the temperature distributions of Al-based MMCs prepared with only a short milling time are shown in Figure 3c,d for comparison. To explain the temperature distribution in the IR images, it is worth noting that uniform distributions of particles lead to uniform thermal conductivity of MMCs; thus, there is a constant temperature gradient in the IR images. There is a uniform temperature distribution in Al-based MMCs prepared with our method, suggesting homogeneous and uniform distributions of SiC and Cu particles in the Al matrix, while there is an obvious temperature difference in Al-based MMCs prepared with a method using a shorter milling time, implying the aggregation of SiC and Cu nanoparticles.

### 3.2. Effect of SiC Addition

**Figure 4:**
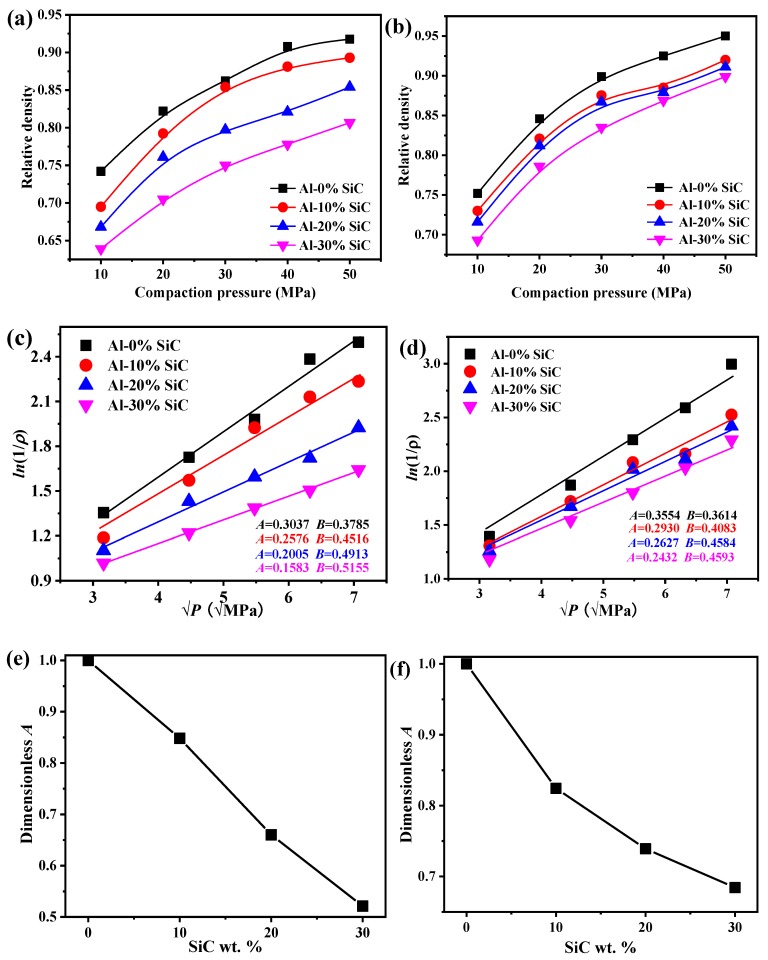
Dependence of compaction pressures on the relative density of Al-SiC MMCs and plastic deformation capacity of Al-SiC MMCs: (**a**) 500-nm Al-SiC MMCs; (**b**) 1-μm Al-SiC MMCs; (**c**) 500-nm Al-SiC MMCs; (**d**) 1-μm Al-SiC MMCs; (**e**) dependence of plastic deformation on weight fraction of 500-nm SiC; (**f**) dependence of plastic deformation on the weight fraction of 1-μm SiC.

The compressibility curves of Al-SiC MMCs prepared with different pressures are shown in Figure 4a,b. It is confirmed that relative density increases with increasing compaction pressure. Adding SiC particles into the Al matrix could improve the compressibility behavior of Al-SiC MMCs, and larger additions of SiC will lead to enhanced compressibility. The enhanced compressibility is a result not only of the high hardness and deformation resistance of SiC, but also of the fragmented SiC particles in the milling process, which may lead to high local internal strains in the composites [43].

To estimate the compressibility of powders, the compaction equation developed by Panelli and Ambrosio [44] was applied to estimate the powder plastic deformation during the cold compaction process:

(2) $$ln\left(\frac{1}{\rho }\right)=A\sqrt{P}+B.$$

where ρ=1−θ, ρ is the porosity, θ is the relative density of MMCs, P is the pressure applied to prepare MMCs, *A* stands for the plastic deformation of the powder, and *B* is the density of powder for the case without loading pressure. Parameters *A* and *B* can be drawn from Figure 4c,d, and correspond to the slope and the truncation of the lines in the figure, respectively. After scaling for different values of *A*, parameters *A* and *B* are further shown in Figure 4e,f. This reveals that the plastic deformation (*A*) decreases with increasing SiC content because of the enhancement of the compression resistance caused by the presence of the hard SiC. Parameter *A* decreases linearly with increasing content of 500-nm MMC, while the decreasing tendency of *A* for 1-μm MMC curves up, indicating that the MMCs are more prone to plastic deformation with 500-nm Al nanoparticles than with microscale Al particles, suggesting an obvious size effect for the elastic modulus. A weight fraction of 30% SiC enhances the compressibility, and this is related to the reciprocal of plastic deformation, which is as high as 100% for 500-nm Al MMCs, while a weight fraction of 30% SiC enhances the compressibility only by about 0.4 times for 1-μm Al MMCs, suggesting that small particles have a greater potential to improve compressibility than large particles, which can be attributed to the size effect of the elastic modulus.

**Figure 5:**
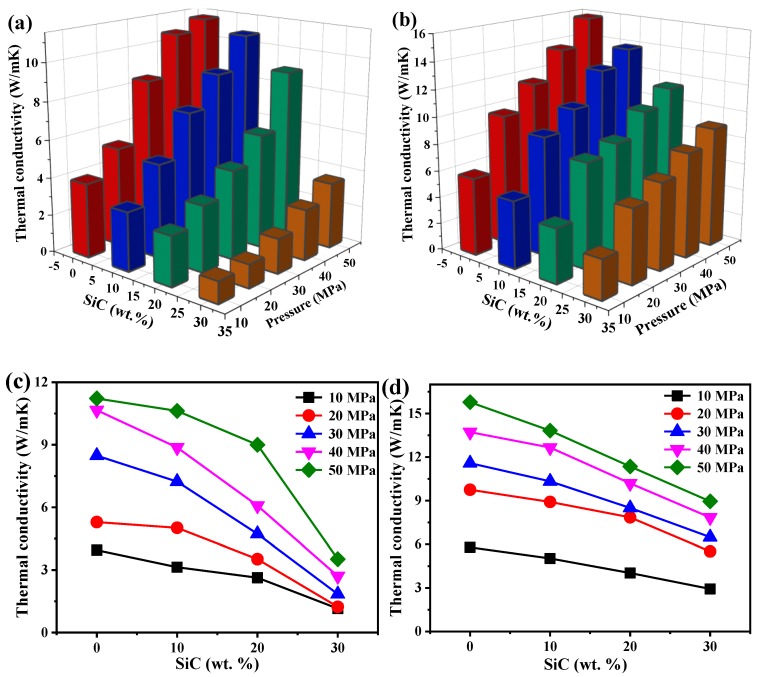
Dependence of thermal conductivity on the weight fraction of SiC and compaction pressure: (**a**) 500-nm Al-SiC MMCs; (**b**) 1-μm Al-SiC MMCs; (**c**) thermal conductivity of 500-nm Al-SiC MMCs; (**d**) thermal conductivity of 1-μm Al-SiC MMCs.

Figure 5a,b shows the dependence of thermal conductivity on the weight fraction of SiC and the compaction pressure. When the weight fraction of SiC is fixed, the thermal conductivity of MMCs increases with increasing compaction pressure, because more packed nanoparticles could result in the deformation of nanoparticles, while also resulting in an enlargement of the contact area between the particles, whereby an enlarged contact area could increase the transportation of electrons and phonons. When the pressure is fixed, the thermal conductivity of Al-SiC MMCs decreases with increasing SiC weight fraction. This can be explained by the fact that the thermal conductivity of SiC particles is lower than that of Al particles, although the thermal conductivity of bulk SiC is higher than that of bulk Al [45], and also that further addition of SiC will cause a greater porosity in MMCs, while a greater porosity significantly hinders electron and phonon transport in MMCs. Figure 5c,d directly displays the relationship between thermal conductivity and SiC weight fraction. With increasing weight fraction, the decreasing trend of thermal conductivity becomes steeper; especially at a compaction pressure of 50 MPa. Similar phenomena for composites were also observed in our theoretical work [46]. Compared with 1-μm Al-SiC MMCs, the thermal conductivity of 500-nm Al-SiC MMCs will be lower, because the heat carrier for SiC is phonons, which can be scattered by smaller sizes.

### 3.3. Effect of Cu Addition

**Figure 6:**
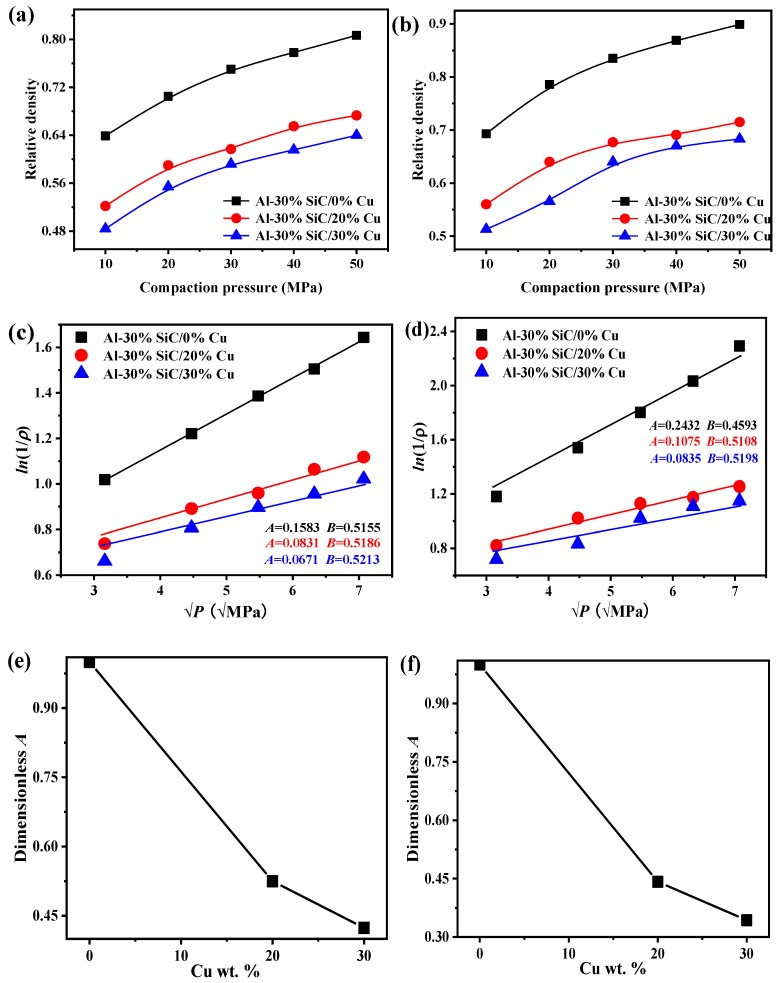
Dependence of compaction pressures on the relative density of Al-SiC/Cu MMCs and plastic deformation capacity of Al-SiC/Cu MMCs: (**a**) 500-nm Al-SiC/Cu MMCs; (**b**) 1-μm Al-SiC/Cu MMCs; (**c**) 500-nm Al-SiC/Cu MMCs; (**d**) 1-μm Al-SiC/Cu MMCs; (**e**) dependence of plastic deformation on Cu weight fraction for 500-nm; (**f**) dependence of plastic deformation on Cu weight fraction for 1-μm.

Cu particles can be further used to enhance the thermal conductivity of Al-SiC MMCs. The influence of Cu additions on the thermal conductivity and compressibility of Al-SiC/Cu MMCs are shown in Figure 6. Similar to what happens for Al-SiC MMCs, the relative density also increases with increasing compaction pressure. Adding Cu particles in Al-SiC MMCs could improve the compressibility behavior of Al-SiC/Cu MMCs, and a larger content of Cu will also lead to an enhanced compressibility, as shown in Figure 6a–d. This enhanced compressibility comes not only from the high hardness and deformation resistance of Cu compared with the Al matrix, but also from the spherical copper nanoparticles, which are able to greatly reduce the contact area between adjacent particles, thus increasing porosity. Figure 6e,f shows that with increasing Cu weight fraction, plastic deformation (*A*) will decrease due to the presence of the hard particles and spherical Cu nanoparticles.

**Figure 7:**
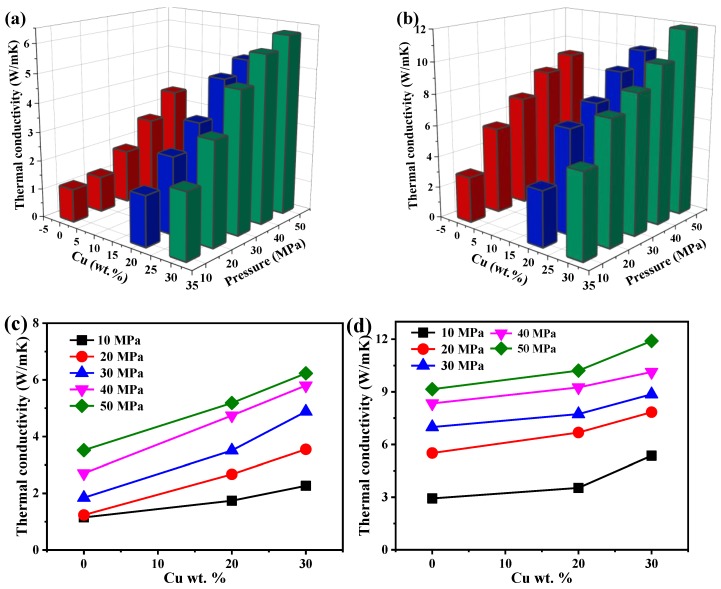
Dependence of thermal conductivity on the weight fraction of Cu and the compaction pressure: (**a**) 500-nm Al-SiC/Cu MMCs; (**b**) 1-μm Al-SiC/Cu MMCs; (**c**) thermal conductivity of 500-nm Al-SiC/Cu MMCs; (**d**) thermal conductivity of 1-μm Al-SiC/Cu MMCs.

Further addition of Cu into Al-SiC further enhances the thermal conductivity, as illustrated in Figure 7. Figure 7a,b shows that increasing both/either the compaction pressure and/or the weight fraction of Cu will lead to enhanced thermal conductivity of Al-SiC/Cu MMCs. This can be explained by the fact that the thermal conductivity of Cu is higher than those of Al and SiC [47,48,49]. Figure 7c,d shows that a higher compaction pressure could enhance the thermal conductivity to more than 2 times that found at low pressure, e.g., for a content of 30%, while the thermal conductivity could only be enhanced by 100% with the increase in content from 0 to 30%. An increase in compaction pressure may be more feasible than adding further reinforcements to enhance the thermal conductivity.

### 3.4. Effect of Sintering Treatment

Although adding Cu can improve the compressive properties and thermal conductivity of Al MMCs, the density of MMCs could also be increased due to Cu having a higher density than Al, which may limit its application. Specific thermal conductivity, which is the ratio of thermal conductivity to mass density, can be used to evaluate the effect of Cu content on these two competition properties (thermal conductivity and mass density). In this section, a sintering treatment is applied in order to increase the specific thermal conductivity of Al MMCs.

**Figure 8:**
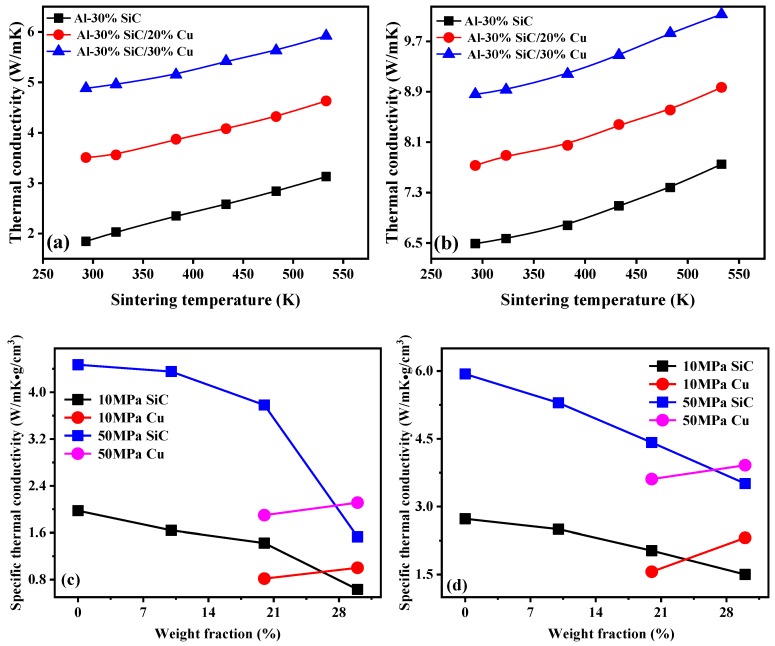

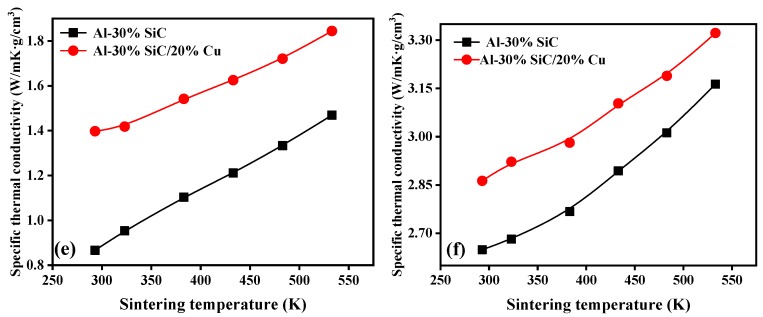
Influences of sintering temperatures and weight fraction of additions on: (**a**) thermal conductivity of 500-nm Al MMCs; (**b**) thermal conductivity of 1-μm Al MMCs; (**c**) specific thermal conductivity of 500-nm Al MMCs; (**d**) specific thermal conductivity of 1-μm Al MMCs; (**e**) specific thermal conductivity of 500-nm Al MMCs; (**f**) specific thermal conductivity of 1-μm Al MMCs.

The effects of sintering temperature on the thermal conductivities of Al MMCs are shown in Figure 8a,b. Thermal conductivity increase with increasing sintering temperature, and higher sintering temperature leads to a higher thermal conductivity, because of the improved interface bonding. More precisely, a higher sintering temperatures will result in better interface contact and will also reduce the grain boundaries on the interface, thus reducing the thermal resistance on the interface [39,41,50,51]. Bending tests of Al MMCs revealed that a higher sintering temperature caused a higher bending strength, which suggests an improved interface bonding and also confirms the possibility of using sintering treatment for enhancing interface bonding, as illustrated in Appendix C. The results for specific thermal conductivity, which depend on the addition content, are shown in Figure 8c,d. The dependence of the specific thermal conductivity on the weight fraction of additions can clearly be seen from the Figure. With increasing compaction pressure, the improvement in thermal conductivity will be greater than of the increase in density, thus resulting in an increase in specific thermal conductivity. The specific thermal conductivity was able to be enhanced by more than 100% with a Cu content of 30% by applying a higher pressure. A larger content of Cu will not greatly increase the specific thermal conductivity, while a larger content of SiC was even able to sharply decrease the specific thermal conductivity. Figure 8e,f shows the dependence of specific thermal conductivity on the sintering temperature. With increasing sintering temperature, the density of the Al MMCs remains almost a constant, while the thermal conductivity increases significantly. Therefore, there is an increase in the specific thermal conductivity, and a higher sintering temperature will lead to a higher specific thermal conductivity. The specific thermal conductivity could be enhanced more than 0.7 times when a sintering temperature of 523 K was applied. To meet the need for low density and high thermal conductivity in applications, it is more desirable to enhance the specific thermal conductivity by enlarging the preparation pressure or/and sintering temperature.

## 4. Conclusions

In this work, utilization of SiC and Cu to enhance the thermal and mechanical properties of Al matrix composites was studied. Firstly, four different weight fractions of SiC were applied in the preparation of Al-SiC powders, and three different weight fractions of Cu were used in the preparation of Al-SiC/Cu powders by ball milling. Subsequently, cold compaction was used to prepare the Al MMCs. The distributions of SiC and Cu in Al MMCs, as characterized by SEM and IR images, confirmed that our ball milling method resulted in homogeneous distributions of SiC and Cu. The mechanical and thermal conductivity measurements revealed that:

(1) A weight fraction of 30% SiC was able to enhance the compressibility by as much as 100% for 500-nm Al MMCs, while a weight fraction of 30% SiC was only able to enhance the compressibility by about 0.4 times for 1-μm Al MMCs, suggesting that small particles have greater potential to improve compressibility than large particles, which can be attributed to the size effect of the elastic modulus. This enhanced compressibility is a result not only of the high hardness and deformation resistance of SiC, but also of the fragmented SiC particles in the milling process, which may lead to high local internal strains in composites. The thermal conductivity of Al-SiC MMCs will decrease with increasing weight fractions of SiC. This can be explained by the fact that the heat carrier for SiC is phonons, which are able to be scattered by smaller sizes.
(2) Adding Cu particles to Al-SiC MMCs improved the compressibility behavior of Al-SiC/Cu MMCs. The enhanced compressibility is a result of the high hardness, high deformation resistance, and the spherical shape of copper nanoparticles, which are able to greatly reduce the contact area between adjacent particles. The thermal conductivity could be enhanced by 100% with an increase in Cu content from 0 to 30%. To meet the need for low density and high thermal conductivity in applications, it is more desirable to enhance the specific thermal conductivity by enlarging the preparation pressure or/and sintering temperature.

## Figures and Tables

**Table 1:** Sizes of raw materials in preparation of Al MMCs.

Raw Materials	Al	SiC	Cu
diameter	500nm or 1 μm	500 nm or 1 μm	500 nm or 1 μm

**Table 2:** Contents of raw materials in Al MMCs.

Prepared Materials	Al-10%SiC	Al-20%SiC	Al-30%SiC	Al-30%SiC/20%Cu	Al-30%SiC/30%Cu
Proportion of SiC in Al	10%	20%	30%	30%	30%
Proportion of Cu in Al-SiC	0	0	0	20%	30%

**Figure A1:**
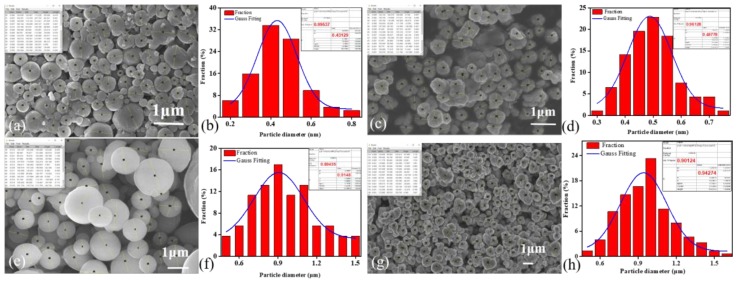
SEM images and size distributions of particles: (**a**,**b**) for 500-nm Al; (**c**,**d**) for 500-nm Cu; (**e**,**f**) for 1-μm Al; (**g**,**h**) for 1-μm Cu.

**Figure A2:**
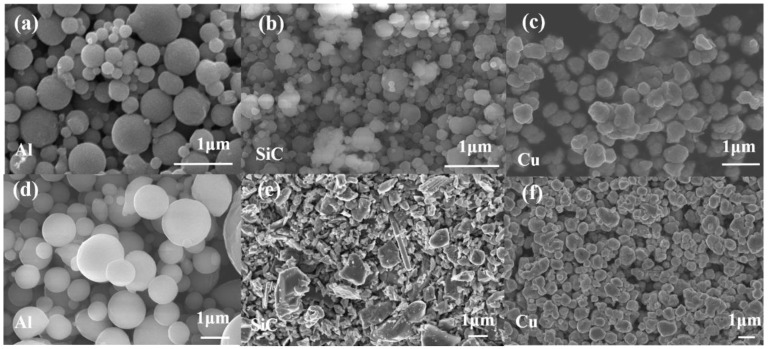
Microstructures of raw particles without ball-milling: (**a**) 500-nm Al; (**b**) 500-nm SiC; (**c**) 500-nm Cu; (**d**) 1-μm Al; (**e**) 1-μm SiC; (**f**) 1-μm Cu.

**Figure A3:**
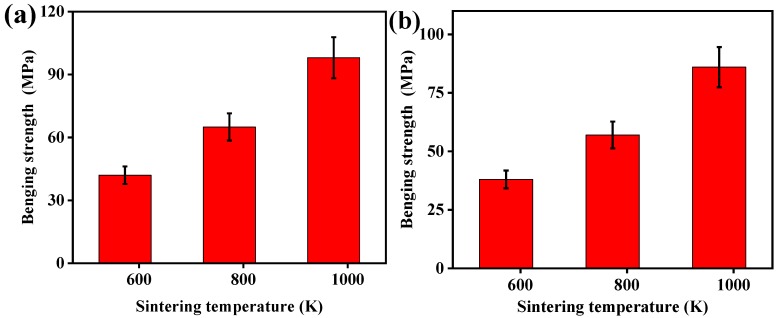
Effects of sintering temperatures on bending strengths of Al MMCs: (**a**) 500-nm Al MMCs; (**b**) 1-μm Al MMCs.

## Appendix A

The average size of particles was calculated from the size distribution of particles obtained from SEM image by ‘Image J’ software.

## Appendix C

Three-point bending strength tests of samples (4 mm × 3 mm × 20 mm) were conducted at a cross-head speed of 0.5 mm/min with a universal testing machine. Results are shown in Figure A3.
